# The Analgesic Effect of Extended Reality (XR) on Acute and Postoperative Pain in Children: A Systematic Review and Meta‐Analysis

**DOI:** 10.1002/pan.70157

**Published:** 2026-03-07

**Authors:** Louise Meulenkamp‐Yilmaz, Souraya El Bardai, Manon H. J. Hillegers, Jeroen Legerstee, Bram Dierckx, Lonneke Staals

**Affiliations:** ^1^ Department of Child and Adolescent Psychiatry/Psychology Erasmus MC Sophia Children's Hospital, University Medical Center Rotterdam Rotterdam the Netherlands; ^2^ Research Institute of Child Development and Education, University of Amsterdam Amsterdam the Netherlands; ^3^ Levvel Mental Health Center Duivendrecht the Netherlands; ^4^ Department of Anesthesiology Erasmus MC Sophia Children's Hospital, University Medical Center Rotterdam Rotterdam the Netherlands

**Keywords:** acute pain, augmented reality (AR), child, extended reality (XR), mixed reality (MR), pediatric patients, postoperative pain, virtual reality (VR)

## Abstract

**Background:**

Acute and postoperative pain in children is often undertreated, with effects on patient comfort and postoperative recovery. Extended reality (XR) interventions offer non‐pharmacological pain management by distracting patients from discomfort. While effective for procedural pain, its impact on prolonged pain episodes remains underexplored.

**Objectives:**

To systematically review and meta‐analyze findings from previous studies on the efficacy of XR interventions in managing acute and postoperative pain in children, compared to standard care.

**Eligibility Criteria:**

Studies involving children (≤ 18 years) with acute or postoperative pain were included if they compared XR interventions to standard care. Studies focusing on procedural or chronic pain were excluded.

**Methods:**

A systematic search was conducted on January 23, 2025, in MEDLINE, EMBASE, Web of Science, CINAHL, and PsycINFO for studies evaluating XR interventions for acute and postoperative pain in children, using validated pain measures. Pain outcomes were extracted for an exploratory meta‐analysis, with self‐report as the primary and observer‐report as the secondary outcome. Two reviewers independently extracted data and assessed study quality using CONSORT and TREND.

**Results:**

From 1793 records, nine studies were included, all evaluating virtual reality (VR) interventions. Seven focused on postoperative pain, two on acute pain. The primary meta‐analysis (*n* = 6) showed a moderate but nonsignificant effect in self‐reported pain (SMD = −0.61; 95% CI, −1.58 to 0.36). The secondary meta‐analysis (*n* = 6) for observer‐reported pain showed a large but nonsignificant effect (SMD = −1.04; 95% CI, −2.18 to 0.11).

**Conclusion:**

This meta‐analysis found no significant analgesic effect of VR on acute or postoperative pain in children. However, moderate effect sizes were observed, but the lack of statistical significance indicates that XR interventions require further investigation in pediatric pain management. Future research should prioritize pain as a primary endpoint and assess the effects of VR type, timing, and age on acute pain using validated measures.

## 1 Introduction

Effective management of acute and postoperative pain in children remains a significant challenge, with pain often undertreated despite advancements in surgical techniques and analgesic options [1, 2]. Children commonly experience pain due to illness or medical procedures. Inadequate pain management can delay recovery, increase anxiety, and elevate the risk of chronic pain, all of which can negatively impact emotional well‐being and development [3, 4, 5]. Moreover, poorly managed pain can lead to fear, trauma, and avoidance of healthcare, further hindering treatment and long‐term health outcomes [5, 6, 7].

While analgesics such as nonsteroidal anti‐inflammatory drugs (NSAIDs) and opioids are commonly prescribed [8], side effects like gastrointestinal issues, respiratory depression, sedation and the potential for addiction are frequent concerns in children [2, 9]. This highlights the need for exploring non‐pharmacological interventions. Emerging techniques like extended reality (XR) [9, 10, 11, 12, 13], video games [14], toys, and music therapy [15] offer effective alternatives for managing procedural pain by diverting attention from discomfort, and may therefore be suitable options for the treatment of acute or postoperative pain.

Immersive or XR technologies are increasingly used in the medical setting, including virtual reality (VR), augmented reality (AR), and mixed reality (MR) [12]. VR fully immerses users in a digital environment, AR overlays digital content onto the real world, and MR integrates both to create interactive experiences [12]. These immersive tools align well with children's cognitive and developmental stages, providing a rich and engaging environment that can capture their attention more effectively than traditional distractions. By immersing children in digital worlds, XR interventions enable them to fully engage, potentially enhancing the pain‐relieving effects [9, 10, 11, 12, 13, 14, 15]. Research indicates that XR technologies effectively reduce anxiety and short‐term procedural pain during medical procedures like blood withdrawal and wound dressing changes by shifting the child's focus from the procedure to nonthreatening stimuli such as interactive games and engaging visuals [9, 10, 11, 12, 13, 14, 15].

While XR has proven effective for short‐term procedural pain, its potential in managing more prolonged pain episodes, such as acute and postoperative pain remains underexplored. This systematic review and meta‐analysis evaluates the analgesic effect of XR interventions for the management of acute and post‐operative pain in children. The review aims to clarify the role of XR in pediatric pain management.

## 2 Methods

A systematic review and meta‐analysis were conducted to evaluate the effectiveness of XR interventions in managing acute and postoperative pain in children. We followed the 2020 PRISMA guidelines to ensure transparency and completeness in the reporting of this review [16]. The PRISMA checklist (6 themes, 27 items) was used to guide the comprehensive reporting process, see Appendix S1. The review protocol was preregistered in PROSPERO, and the study is registered on the Open Science Framework (OSF) (https://doi.org/10.17605/OSF.IO/CW9QN).

### 2.1 Selection Criteria

We included intervention studies, both randomized and non‐randomized, that compared an XR intervention group (using VR, AR, MR, or 360° video) with a control group receiving standard or usual care. Studies were eligible if they reported on children or adolescents up to 18 years old experiencing acute or postoperative pain, and if pain was measured using validated age‐appropriate instruments. Studies were excluded if they lacked a control group, focused on procedural or chronic pain (pain persisting for at least three months), or did not provide extractable pain outcomes. Studies with missing data were excluded if authors did not respond to data requests. For the purposes of this review, acute pain is defined as pain that arises suddenly due to injury, surgery, illness, or trauma, typically of limited duration (usually less than 7 days, but it may extend up to 30 days) (International Association for the Study of Pain (IASP)) [8]. In this review, postoperative pain was explicitly mentioned as a search criterium, and defined as acute pain following surgical procedures [8].

### 2.2 Search Strategy

An exhaustive search was conducted by a biomedical information specialist on [2024/11/04] to identify relevant articles published in English. The search encompassed the following electronic databases: MEDLINE, EMBASE, Web of Science, CINAHL, and PsycINFO. The search strategy employed specific terms related to XR, VR including 360° videos, AR, and MR, and acute and postoperative pain, focusing on “infant,” “child,” and “adolescent” populations. Different search strategies tailored to each database were developed to ensure comprehensive coverage of the literature. An update of the search was performed on January 23, 2025. A detailed overview of the search strategy is provided in Appendix S2.

### 2.3 Study Selection

The study selection and data extraction were conducted using Covidence (Veritas Health Innovation, Melbourne, Australia) [17]. Data were reviewed independently by two authors (L.M. and S.E.B.) to ensure accuracy and consistency. Reviewers reached initial agreement on 75% of full‐text assessments; remaining discrepancies (25%) were resolved through discussion. In some cases, particularly when there was uncertainty about study eligibility or the appropriate timing of VR intervention relative to pain assessment, consensus was reached in consultation with an anesthesiologist (L.S.). Data analysis was conducted using IBM SPSS Statistics (version 28) by two authors (L.M. and B.D.). A meta‐analysis was performed when data from at least five studies were available.

### 2.4 Data Extraction

To capture relevant information from all included studies, the following parameters were extracted if provided:

– Study ID: reference, first author, title, year, journal/source and country of origin.
– Study methods: design, (validated/unvalidated) pain measurements instruments, self reported pain, or reported by others (parent, caregiver, health care professional).
– Study participants: setting, sample size, age, gender, medical condition, effect on pain scores, pain severity (mild, moderate, severe) if available; type of pain (i.e., acute, postoperative, oncological or other), pain medication (analgesics), patient satisfaction, user experience of XR.
– Study intervention: XR type, intervention content, duration and frequency of XR, timing of XR application and characteristics of the control group condition.
– Study measure: main and secondary outcomes, adverse effects such as nausea and dizziness (if reported).

Means and standard deviations (SD) were extracted where available. If these were not provided, median and interquartile range (IQR) data were used. These were then converted into means and SDs using the method by [18] to facilitate comparison and analysis.

### 2.5 Assessment of Study Quality

For the assessment of study quality in this systematic review, we used specific checklists corresponding to the study design of each included study. Randomized Controlled Trials (RCTs) were evaluated using the CONSORT 2025 (Consolidated Standards of Reporting Trials) checklist, which consists of 30 items [19]. This version includes refinements to existing items and new guidance on transparency, implementation, and equity‐related reporting. For non‐randomized but controlled trials, the TREND (Transparent Reporting of Evaluations with Nonrandomized Designs) checklist was used, consisting of 22 items [20]. Since these tools assess different study designs, the scores are not directly comparable; however, for clarity, we expressed adherence to each checklist as a percentage of the total applicable criteria met.

For CONSORT, a maximum score for each RCT was calculated by subtracting the number of not applicable (NA) items from the theoretical maximum of 30. Each item was equally weighted, with 0.5 points deducted for partially reported items when applicable. Similarly, for the TREND statement, a maximum score was determined by excluding nonrelevant items, and the percentage compliance was calculated by dividing the total score by the adjusted maximum.

### 2.6 Synthesis of Results

The primary analysis focused on self‐reported pain scores from children, while the secondary analysis included observer‐reported pain scores, assessed by parents or healthcare professionals.

Initially, we aimed to analyze acute and postoperative pain separately. However, due to the limited number of acute pain studies, these outcomes were pooled. To explore potential sources of heterogeneity, sensitivity analyses were conducted based on pain type (acute vs. postoperative) and pain assessor (self‐reported vs. observer‐rated).

Standardized mean differences (SMDs) were calculated to quantify XR's effect on pain, by dividing the difference in pain scores between XR and control groups by the pooled standard deviation (SD) [18]. Cohen's rule of thumb was used to interpret the SMD: 0.2 was considered a small effect, 0.5 medium effect, and 0.8 large effect [21]. A random‐effects model was applied to account for heterogeneity due to variation in pain measurement instruments, XR interventions, and pain types (acute vs. postoperative). Heterogeneity was assessed using the *I* ^2^ statistic, with values ≥ 75% indicating substantial heterogeneity [22]. Publication bias was assessed using funnel plots [23].

## 3 Results

**FIGURE 1:**
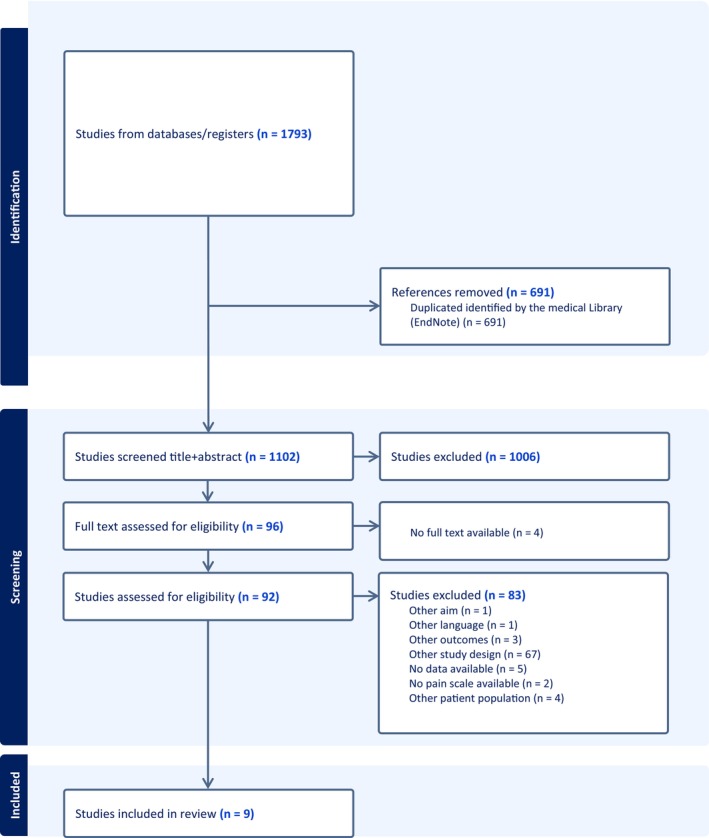
PRISMA flowchart.

The initial search yielded 1793 records, of which 1102 remained after duplicate removal. Following title and abstract screening, 96 full‐text articles were assessed, and 83 were excluded (including four without accessible full texts). An updated search on 23 January 2025 identified two additional eligible studies, resulting in a total of nine studies included in the meta‐analysis (Figure 1). The nine studies examined acute pain (*n* = 2) and postoperative pain (*n* = 7) across various surgeries. The evidence comprised eight randomized controlled trials and one quasi‐experimental pilot study. The sample included 1042 children aged 3–18 years. The characteristics and outcomes of the included studies, published between 2019 and 2024, are summarized in Table 1.

**TABLE 1:** Characteristics and results of included studies.

Author/year	Number of participants	Study design	Participant age group	VR equipment	Timing of VR	Intervention application	Care as usual	Outcome measure(s)	Type of pain	Key findings
Binay et al. 2022	132	RCT	6–12 years	VR goggles	Before general or day surgery	Educational Animation Group; Animated movie in a 3‐Dimensional environment which included all routine procedures of preoperative preparation and postoperative for the child and the parent in the pediatric surgery clinic (*n* = 44)	Care as usual (*n* = 44)	WBFPRS	Post‐operative	The study found that the VR intervention significantly reduced postoperative pain in pediatric patients (VR: mean 5.04, SD 0.66; control: mean 7.85, SD 1.11; *p* < 0.001), with the WBFPRS showing a reduction as reported by the child
Buyuk et al. 2021	78	RCT	5–10 years	A white‐colored VR BOX 3.000 with VR glasses (a headset with a screen covering)	Before circumcision surgery	In the VR program named Amazon, the child perceives him/herself to be walking among the trees in the Amazon forests. The other VR program gives the child a feeling of water skiing (*n* = 40)	Care as usual (*n* = 38)	WBFPRS	Post‐operative	The study found that the VR intervention before surgery significantly reduced postoperative pain in pediatric patients (VR: mean 1.18, SD 0.75; control: mean 3.16, SD 1.38; *p* < 0.001), with the WBFPRS showing a reduction as reported by parents
Eijlers et al. 2019	191	RCT	4–12 years	HTC Vive (HTC Corporation, Xindian, New Taipei, Taiwan) head‐mounted display	Before Adenoidectomy and/or tonsillectomy, tympanostomy tubes, maxillofacial and dental procedures other ENT surgeries	VRE exposed to a realistic child‐friendly immersive virtual version of the operating theater (*n* = 94). Accompanying parents can watch the video on a laptop at the same time, so that they can see what their children are watching	Care as usual (*n* = 97)	FPS‐R FLACC	Post‐operative	The study found no significant differences in postoperative pain levels between the VRE and control groups (VR: mean 1.92, SD 2.62; control: mean 1.92, SD 2.62; *p* = 1.00), as measured by the FPS‐R reported by the child
Specht et al. 2023	106	RCT	7–18 years	The Oculus Go (Irvine, CA) headset was preloaded with the Nature Treks VR application which provides immersive audio‐visual environments to explore with different settings ranging from outer space to the deep sea	Before general, spine, other orthopedic, or burn surgery	The Nature Treks VR application provides children with immersive audio‐visual environments to explore with different settings ranging from outer space to the deep sea (*n* = 50)	iPad 5 (Cupertino, CA), or newer, for postoperative distraction. These tablet devices are preloaded with educational games approved by UNC Child Life Specialists to appeal to a wide range of ages and interests (*n* = 56)	WBFPRS FLACC VAS	Post‐operative	The study found no significant differences in postoperative pain levels between the VR and control groups (VR: mean 0.548, SD 1.71; control: mean 0.487, SD 1.12; *p* = 0.857), as measured by the FLACC, reported by the researcher
Wu et al. 2022	99	RCT	6–9 years	A Head‐mounted display (CV1 PRO, NOLO VR, Beijing, China)	Before ENT, penoplasty or other surgery	The VR tool comprises a highly realistic virtual environment modeled according to the real operating room and medical personnel. The virtual environment is computer generated, interactive and child friendly. Accompanying parents can watch the video on a laptop at the same time, so that they can see what their children are watching (*n* = 54)	Care as usual (*n* = 54)	FLACC	Post‐operative	The study found no significant differences in postoperative pain levels between the VR and control groups (VR: mean 0.333, SD 0.76; control: mean 0.333, SD 0.76; *p* = 1.00), as measured by the FLACC, reported by the nurse
Carbó et al. 2024	241	RCT	3–12 years	VR headset (Samsung Gear VR, Samsung Electronics, Seoul, South Korea)	Before OST, general, maxillofacial, ophthalmology, ENT or combined surgery	Children in the intervention group viewed a VR‐based educational video on the surgical process (*n* = 120)	Care as usual (*n* = 121)	WBFPRS	Post‐operative pain	The study found that the VR intervention before surgery significantly reduced postoperative pain, with lower pain scores in the VR group compared to the control group (VR: mean 0.2, SD 0.6; control: mean 0.6, SD 0.9; *p* < 0.001), as measured by the WBFPRS, reported by the child
Turgut et al. 2024	70	RCT	4–10 years	VR glasses (Oculus Go VR) The recordings of the tour video were taken with a Samsung Gear 360 camera	Before endoscopy, hernia, hydrocele, hypospadias, undescended testicle, double J‐stent, cyst excision, or circumcision surgery (on the day of surgery and at least one hour before surgery) Postoperative (Following their surgery, children in the VR group also watched 360‐degree cartoons of their choice during the first nursing procedures after the effect of the anesthesia wore off)	Children in the VR group watched a 360‐degree video tour of the entire operating theater area for one‐and‐a‐half‐minute. In this video, a nurse explained the preoperative and postoperative processes for the day of the surgery (*n* = 35)	Care as Usual (*n* = 35)	WBFPRS	Post‐operative pain	The study found no significant differences in postoperative pain levels between the VR and control groups (VR: mean 3.2, SD 0.99; control: mean 3.46, SD 1.07; *p* = 0.300), as measured by the WBFPRS, reported by the child
Butt et al. 2022	110	RCT	13–17 years	The Oculus Go headset (Facebook Technologies LLC., released 2018, Oculus Go, Android 7.1.2), which had the Take‐Pause software predownloaded by Take Pause Inc.	During acute pain episode (emergency department)	Children were given 2 min to become accustomed with the headset and make necessary adjustments according to their level of comfort. Once familiar with the device, the child logged onto the Take‐Pause software and participated in the mindfulness exercise for 3 min (*n* = 55)	Patients were allowed to play any game or games of their choice on an iPad for 5 min. The iPad was predownloaded with age‐appropriate gaming applications from the Apple App store (Apple Inc., iOS 10.2, updated 2017) (*n* = 55)	WBFPRS	Acute pain	The study found no significant reduction in pain scores between the VR and iPad groups, as there was no statistically significant difference in pain levels post‐intervention (VR: mean 3.58, SD 3.1; control: mean 3.55, SD 3.3; *p* = 0.690), as measured by the WBFPRS, reported by the child
Diaz et al. 2019	15	Pilot study (quasi‐experimental)	8–18 years	The Google Daydream VR. The VR consisted of a headset that wears like a pair of goggles and a screen from a Google Pixel cell phone inside the headset	During acute pain episode (emergency department)	VR for 15 min after intravenous administration (*n* = 8). The VR interventions included applications such as Wonderglade (interactive mini‐games), Ocean Rift (ocean exploration), Karts Sprint (car racing), Ace Fishing (3D fishing adventure), and The Turning Forest (storytelling with a magical forest). The headset blocked the participant's view of their real surroundings	Standard care at the acute care hospital consisted of intravenous narcotics every 30 min as needed for up to three doses (*n* = 7)	NRS FLACC	Acute pain	The study found no significant reduction in observed pain scores compared to the control group (VR: mean 5.71, SD 2.75; control: mean 5.25, SD 2.25; *p* = 0.73), as measured by the FLACC scale, reported by the child

Abbreviations: ENT, ear, nose and throat; FLACC, Face, Legs, Activity, Cry, Consolability; FPS‐R, Faces pain Scale‐Revised; NRS, Numeric Rating Scale; OST, orthopedic surgery and/or traumatology; RCT, randomized controlled trial; SD, standard deviation; VAS, Visual Analog Scale; WBFPRS, Wong‐Baker Faces Pain Rating Scale.

VR distraction was used for acute pain [24], while interactive VR games targeted acute pain in sickle cell crisis specifically [25], both in the emergency department. Postoperative pain studies covered a range of surgeries, including urological, ENT, orthopedic, and others [26, 27, 28, 29, 30, 31, 32]. However, it is important to note that most of these studies primarily used VR for preoperative education and preparation. These preoperative VR interventions aimed to reduce pain and anxiety by providing an immersive experience of the surgical process, such as an immersive VR version of the operating theater [26, 28, 30, 31, 32]. A few studies used VR for immersive distraction, with programs such as *Amazon* (featuring activities like water skiing and forest walking) [27] and *Nature Treks* (exploring diverse environments) [29].

Control groups received CAU, which varied across studies. In acute pain settings, CAU included passive tablet‐based distraction [24] or standard pharmacological management for sickle cell crisis [25]. In postoperative studies, CAU generally consisted of conventional preoperative education delivered verbally, in writing, or both [26, 27, 28, 30, 31, 32], with some protocols limited to written information and consent only [32] or waiting without parental presence [31]. One study provided non‐immersive tablet games unrelated to analgesia [29].

All studies used validated, age‐appropriate pain assessment tools, including the Wong‐Baker Faces Pain Rating Scale (WBFPRS) [24, 26, 27, 29, 31, 32], Faces Pain Scale‐Revised (FPS‐R) [28], Numerical Rating Scale (NRS) [25], Visual Analog Scale (VAS) [29], and the FLACC scale [25, 28, 29, 30]. The WBFPRS and FPS‐R were primarily used for younger children, while the NRS and VAS were applied to older children.

### 3.1 Study Quality Assessment

The CONSORT 2025 checklist applied to RCTs, scores ranged from 72% to 82%, with an average of 76%. Common limitations included missing allocation concealment, lack of access to full protocols, and incomplete reporting of harms. For the TREND checklist, used for non‐randomized studies, scores ranged from 63% to 70%, with common gaps including missing allocation concealment, trial registration, group analyses, and harms reporting. See Table 2. Despite these gaps, most studies provided sufficient methodological details to ensure transparency and reproducibility.

**TABLE 2:** Quality assessment.

Autho**r**	Checklist	Quality‐score	Reporting gaps
Binay et al. 2022	CONSORT	78%	Minor gaps: allocation concealment and additional analyses. No report of allocation concealment
Buyuk et al. 2021	CONSORT	72%	Minor gaps: title missing “RCT,” missing the protocol and statistical analyses and additional analyses. Also missing the descript of changes to trial protocol, reporting harms, stopping guidelines, details of patient in the design and the blinding method
Eijlers et al. 2019	CONSORT	75%	Missing protocol access and harms reporting, no data sharing, blinding details, patient involvement, or post hoc analysis description. Allocation concealment is partly clarified
Specht et al. 2023	CONSORT	80%	Minor gaps: title missing “RCT,” additional analyses method not described, no data sharing statement, no harms explanation, unclear allocation concealment and handling of missing data. Strengths: clear blinding and randomization grouping
Wu et al. 2019	CONSORT	75%	Missing recruitment dates, data sharing, patient involvement, and pre‐specified analyses; harms were reported, but not defined. Strengths: clear blinding, group allocation, and handling of missing data
Carbó et al. 2024	CONSORT	82%	Missing allocation concealment and protocol access and harms reporting (low‐risk VR intervention)
Turgut et al. 2024	CONSORT	74%	Missing protocol access and harms reporting, no data sharing, blinding details, patient involvement, or post hoc analysis description. Allocation concealment and adherence/fidelity only partially addressed. No reporting on concomitant care or missing data handling
Butt et al. 2022	TREND	70%	Missing allocation concealment, trial registration, group analyses, protocol access, and harms reporting (low‐risk VR intervention). Open science not described
Diaz et al. 2019	TREND	63%	Pilot study; missing allocation concealment and detailed analysis methods. Nothing is mentioned about the blinding and the handling of missing data. Open science is not described

### 3.2 Extended Reality and Pain Management

Six of the nine studies were included in both the primary and secondary exploratory meta‐analyses (*n* = 6), with three studies reporting both self‐reported and observer‐reported pain. Data from both acute [24, 25] and postoperative pain [26, 27, 28, 29, 30, 31, 32] were combined. Given the limited number of acute pain studies (*n* = 2) and the presence of an outlying study in the postoperative pain group [26], a sensitivity analysis was conducted to assess the robustness of findings for both acute and postoperative pain. Study characteristics of included trials are summarized in Table 1.

### 3.3 Meta‐Analysis

The primary meta‐analysis (*n* = 6) on self‐reported pain showed a moderate weighted effect size (SMD = −0.61; 95% CI, −1.582 to 0.360; *p* = 0.22, *I* ^2^ = 0.97), indicating no statistically significant difference between the XR intervention and CAU. The secondary meta‐analysis (*n* = 6) on observer‐reported pain showed a large weighted effect size (SMD = −1.04; 95% CI, −2.18 to 0.11; *p* = 0.08, *I* ^2^ = 0.97), but the result was not statistically significant. All corresponding forest plots for these analyses are presented in Appendix S3.

### 3.4 Sensitivity and Subgroup Analyses

High heterogeneity was observed in both primary and secondary analyses (*I* ^2^ = 97%), reflecting substantial methodological variability across studies. To explore this, sensitivity analyses were performed excluding one study [26] that reported both patient‐ and observer‐reported pain outcomes and therefore overlapped across both analyses.

Exclusion of study 24 reduced heterogeneity and yielded a smaller, still nonsignificant effect in the primary analysis (SMD = −0.18; 95% CI, −0.44 to 0.09; *p* = 0.18; *I* ^2^ = 0.57). In the secondary analysis, exclusion of study 24 resulted in a larger, yet still nonsignificant effect (SMD = −0.52; 95% CI, −1.25 to 0.22; *p* = 0.17; *I* ^2^ = 0.92).

Subgroup analysis for acute pain was not feasible due to an insufficient number of studies. For postoperative pain, pooled exploratory analyses showed marked differences for patient‐ and observer‐reported outcomes. With all studies included, the effect size for patient‐reported pain was SMD = −0.18 (95% CI, −0.52 to 0.89; *p* = 0.61; *I* ^2^ = 0.94), while the observer‐reported effect was SMD = −1.04 (95% CI, −2.43 to 0.34; *p* = 0.14; *I* ^2^ = 0.98).

To further explore these findings, postoperative subgroup sensitivity analyses were conducted excluding study 24. For the patient‐reported outcome, the effect size was SMD = −0.26 (95% CI, −0.60 to 0.07; *p* = 0.12; *I* ^2^ = 0.69), and for the observer‐reported outcome, SMD = −0.52 (95% CI, −1.25 to 0.22; *p* = 0.17; *I* ^2^ = 0.92). Although heterogeneity was reduced, results remained nonsignificant and inconsistent. All corresponding forest plots for these analyses are presented in Appendix S3.

### 3.5 Publication Bias

The funnel plots shows some asymmetry, but with the small number of included studies it is difficult to draw firm conclusions about publication bias, see Appendix S4. The observed asymmetry could be due to true heterogeneity between studies, random variation, or potential publication bias. Given the limited number of studies, further statistical tests such as Egger's test or trim‐and‐fill were not performed.

## 4 Discussion

This systematic review and exploratory meta‐analysis examined whether XR has an analgesic effect on acute and postoperative pain in children, based on nine studies with a total of 1042 participants. No statistically significant reduction in self‐reported pain was found, while observer‐reported outcomes showed moderate but nonsignificant effect sizes [24, 25, 26, 27, 28, 29, 30, 31, 32]. Given the limited number of studies, high heterogeneity of the data, and diverse interventions used, the results should be interpreted with caution.

The observed heterogeneity is likely due to several factors. In all but three studies [25, 26, 28], pain was assessed as a secondary outcome and studies were primarily powered for anxiety or preparatory effects, limiting the ability to detect clinically meaningful analgesic effects (> 1 point pain reduction). Additionally, the lack of acute pain studies involving XR interventions and the predominance of preoperative VR interventions (with only one postoperative study using VR after surgery, rather than only before) [29] contributed to the observed heterogeneity. The use of varied VR technologies, ranging from 360° videos to immersive VR, also added to the variability in results.

Despite these differences, the interventions employed in these studies share a common underlying technology‐based approach. Pooling these interventions allowed for an evaluation of XR as a broad class of non‐pharmacological analgesic interventions, rather than focusing on any single intervention. This broader exploratory approach provides valuable insights into the potential of XR technologies in pediatric pain management.

Although clinical heterogeneity raises concerns about the appropriateness of combining such diverse studies in a meta‐analysis, it is important to note that conducting a meta‐analysis is justified when the aim is to explore the overall effectiveness of an intervention across diverse real‐world settings. In this case, a random‐effects model was employed to account for variability between studies, and heterogeneity was explicitly reported to acknowledge that true effects may vary across different contexts.

Synthesis of heterogeneous evidence in this way is particularly important in emerging fields, such as pediatric XR‐based pain management, where studies are often small, context‐specific, and lack uniformity. Pooling the data increases statistical power, enhances the precision of effect estimates, and helps identify consistent patterns of benefit. This approach not only highlights trends in the existing body of research but also lays the groundwork for future, more targeted studies to further refine the understanding of XR's role in pediatric pain management.

However, as with many emerging fields, variability in study design, including differences in pain assessment timing, informants, and context, presents a challenge for interpreting findings. Most of the postoperative studies measured pain shortly after surgery, often during nursing care or in the recovery ward [26, 27, 28, 30, 31, 32]. Additionally, the majority of these studies primarily applied VR preoperatively to reduce anxiety and prepare patients for surgery, rather than using it to address postoperative pain [26, 28, 30, 31, 32]. Only one study applied VR after surgery during the pain episode itself, with repeated short‐interval pain measures, yet reported no analgesic effect [29].

A recent study [33] further supports the impact of timing, showing that VR interventions given both before and after surgery may lead to different outcomes, depending on when they are administered. VR content ranged from relaxing or educational to interactive distraction, yet analgesic co‐interventions were inconsistently documented, limiting interpretation of VR's additive effect [25, 29, 31]. One high‐quality outlier study (78% quality score) reported large effects, suggesting that timing of VR exposure and participant characteristics, such as type of surgery or pain history, may influence outcomes [26]. The inclusion of diverse VR technologies (e.g., immersive VR vs. 360° video) may have different effects on pain relief and potentially cause cybersickness [34], but this was not consistently addressed in the included studies. In acute pain settings, VR was applied briefly and primarily for distraction [24, 25]. Notably, Diaz‐Hennessey et al.'s [25] study focused on children with sickle cell disease, whose recurrent pain and coping mechanisms may differ from children with first‐time acute pain, highlighting the need to explore in future studies pain history as a moderating factor.

Pain was typically assessed shortly after intervention, potentially missing delayed effects. As some interventions may exert cumulative or longer‐term benefits, immediate assessments alone could underestimate their true impact. Observer‐reported pain reductions were generally larger than self‐reported ones, although the use of VR headsets may obscure behavioral pain cues critical for accurate observer ratings, potentially biasing results; therefore, self‐report remains the gold standard, especially in children aged six and older [24, 25, 26, 29].

The findings of this review highlight the need for further focused research on XR interventions for pain management in pediatric populations. While XR shows potential in some studies, its overall effectiveness remains inconclusive at the individual level. Future studies should investigate the effects of XR based on the type of pain (acute vs. postoperative), the timing of interventions (preoperative vs. postoperative), and the specific XR technology used (immersive vs. 360‐degree VR). A notable gap exists in the literature regarding XR's impact on acute pain, and many postoperative studies have focused more on anxiety reduction rather than pain management. Furthermore, more research is needed to explore adverse events like cybersickness and their impact on postoperative pain. To enhance future studies, pain reduction should be prioritized as the primary outcome, with consistent reporting across diverse patient populations and interventions.

In conclusion, current evidence does not conclusively support XR's analgesic efficacy in acute or postoperative pain in children. This highlights the urgent need for well‐powered, methodologically rigorous trials that focus on pain as a primary outcome. Additionally, future studies should evaluate XR interventions during active pain episodes rather than only preparatory use.

Additional resources, including data tables, search strategies, and further analyses, are presented in the Supporting Information.

## Supporting information


**Appendix S1:** pan70157‐sup‐0001‐AppendixS1.docx.

**Appendix S2:** pan70157‐sup‐0002‐AppendixS2.docx.

**Appendix S3:** pan70157‐sup‐0003‐AppendixS3.docx.

**Appendix S4:** pan70157‐sup‐0004‐AppendixS4.docx.

## Data Availability

The data that support the findings of this study are available from the corresponding author upon reasonable request.
