# Supplementary material for: The Analgesic Effect of Extended Reality (XR) on Acute and Postoperative Pain in Children: A Systematic Review and Meta‐Analysis
Source: Paediatr Anaesth. 2026 Mar 7;36(5):479–90. doi: 10.1002/pan.70157 (PMC13054109; doi:10.1002/pan.70157)
Supplement: Supplementary file 2 — Appendix S2: pan70157‐sup‐0002‐AppendixS2.docx. [file PAN-36-479-s002.docx]

# ***Appendix S2: Search strategy***

| Database searched | Platform | Years of coverage | Records | Records after duplicates removed |
| --- | --- | --- | --- | --- |
| Medline ALL | Ovid | 1946 - Present | 370 | 366 |
| Embase | Embase.com | 1971 - Present | 648 | 404 |
| Web of Science Core Collection* | Web of Knowledge | 1975 - Present | 554 | 267 |
| CINAHL Plus | EBSCO | 1982 - Present | 127 | 26 |
| PsycINFO | Ovid | 1806 - Present | 94 | 39 |
| Total | | | 1793 | 1102 |

*Science Citation Index Expanded (1975-present) ; Social Sciences Citation Index (1975-present) ; Arts & Humanities Citation Index (1975-present) ; Conference Proceedings Citation Index- Science (1990-present) ; Conference Proceedings Citation Index- Social Science & Humanities (1990-present) ; Emerging Sources Citation Index (2005-present). No other database limits were used than those specified in the search strategies.

**Medline**

(Virtual Reality / OR Virtual Reality Exposure Therapy / OR Augmented Reality / OR Exergaming / OR (((virtual* OR augment* OR extended* OR mixed*) ADJ3 realit*) OR (virtual* ADJ3 anesthe*) OR exergam* OR vr OR (360 ADJ3 video*)).ab,ti,kw.) AND (Pain/ OR Pain Measurement/ OR exp Analgesia/ OR exp Anesthesia / OR (pain* OR analges* OR anesthe* OR anaesthe*).ab,ti,kw.) AND (exp Child / OR exp Infant/ OR Adolescent / OR Pediatrics / OR (child* OR infan* OR adolescen* OR pediatric* OR paediatric*).ab,ti,kw.) NOT (* "Wounds and Injuries "/ OR * Pain, Procedural / OR exp * Burns / OR exp * Bandages / OR (wound* OR procedur* OR periprocedur* OR needle* OR burn* OR labor* OR labour* OR obstetric* OR injection* OR intravenous* OR cannul* OR chemotherap* OR treatment* OR puncture* OR venipuncture*).ti.)

**Embase**

('virtual reality'/de OR 'virtual reality exposure therapy'/de OR 'augmented reality'/exp OR 'augmented reality system'/exp OR 'virtual reality system'/exp OR exergaming/de OR 'extended reality'/de OR 'mixed reality'/de OR (((virtual* OR augment* OR extended* OR mixed*) NEAR/3 realit*) OR (virtual* NEAR/3 anesthe*) OR exergam* OR vr OR (360 NEAR/3 video*)):Ab,ti,kw) AND (pain/exp OR 'pain parameters'/exp OR 'pain measurement'/de OR 'pain assessment'/exp OR analgesia/exp OR anesthesia/exp OR (pain* OR analges* OR anesthe* OR anaesthe*):ab,ti,kw) AND (child/exp OR adolescent/exp OR childhood/exp OR adolescence/exp OR pediatrics/exp OR 'pediatric patient'/de OR (child* OR infan* OR adolescen* OR pediatric* OR paediatric*):ab,ti,kw) NOT ('wound care'/mj/exp OR 'procedural pain'/exp/mj OR procedures/mj OR 'medical procedures'/mj OR 'invasive procedure'/mj OR 'burn patient'/mj OR 'bandages and dressings'/exp/mj OR (wound* OR procedur* OR periprocedur* OR needle* OR burn* OR labor* OR labour* OR obstetric* OR injection* OR intravenous* OR cannul* OR chemotherap* OR treatment* OR puncture* OR venipuncture*):ti)

**Web of science**

TS=((((virtual* OR augment* OR extended* OR mixed*) NEAR/2 realit*) OR (virtual* NEAR/2 anesthe*) OR exergam* OR vr OR (360 NEAR/2 video*))) AND TS=((pain* OR analges* OR anesthe* OR anaesthe*)) AND TS=((child* OR infan* OR adolescen* OR pediatric* OR paediatric*)) NOT TI=((wound* OR procedur* OR periprocedur* OR needle* OR burn* OR labor* OR labour* OR obstetric* OR injection* OR intravenous* OR cannul* OR chemotherap* OR treatment* OR puncture* OR venipuncture*))

**CINAHL**

(MH Virtual Reality OR MH Virtual Reality Exposure Therapy OR MH Augmented Reality OR MH Exergames OR TI(((virtual* OR augment* OR extended* OR mixed*) N2 realit*) OR (virtual* N2 anesthe*) OR exergam* OR vr OR (360 N2 video*)) OR AB(((virtual* OR augment* OR extended* OR mixed*) N2 realit*) OR (virtual* N2 anesthe*) OR exergam* OR vr OR (360 N2 video*))) AND (MH Pain + OR MH Pain Measurement OR MH Analgesia+ OR MH Anesthesia + OR TI(pain* OR analges* OR anesthe* OR anaesthe*) OR AB(pain* OR analges* OR anesthe* OR anaesthe*)) AND (MH Child + OR MH Infant+ OR MH Adolescent + OR MH Pediatrics + OR TI(child* OR infan* OR adolescen* OR pediatric* OR paediatric*) OR AB(child* OR infan* OR adolescen* OR pediatric* OR paediatric*)) NOT (MM "Wounds and Injuries +" OR MM Pain, Procedural OR MM Burns + OR MM " Bandages and Dressings +" OR TI(wound* OR procedur* OR periprocedur* OR needle* OR burn* OR labor* OR labour* OR obstetric* OR injection* OR intravenous* OR cannul* OR chemotherap* OR treatment* OR puncture* OR venipuncture*))

**PsycINFO**

(Virtual Reality / OR Augmented Reality / OR (((virtual* OR augment* OR extended* OR mixed*) ADJ3 realit*) OR (virtual* ADJ3 anesthe*) OR exergam* OR vr OR (360 ADJ3 video*)).ab,ti.) AND (Pain/ OR Pain Measurement/ OR exp Analgesia/ OR exp Anesthesia / OR (pain* OR analges* OR anesthe* OR anaesthe*).ab,ti.) AND (100.ag. OR 200.ag. OR Pediatrics / OR (child* OR infan* OR adolescen* OR pediatric* OR paediatric*).ab,ti.) NOT (* Wounds / OR exp * Burns / OR (wound* OR procedur* OR periprocedur* OR needle* OR burn* OR labor* OR labour* OR obstetric* OR injection* OR intravenous* OR cannul* OR chemotherap* OR treatment* OR puncture* OR venipuncture*).ti.)
