# Supplementary material for: The Analgesic Effect of Extended Reality (XR) on Acute and Postoperative Pain in Children: A Systematic Review and Meta‐Analysis
Source: Paediatr Anaesth. 2026 Mar 7;36(5):479–90. doi: 10.1002/pan.70157 (PMC13054109; doi:10.1002/pan.70157)
Supplement: Supplementary file 3 — Appendix S3: pan70157‐sup‐0003‐AppendixS3.docx. [file PAN-36-479-s001.docx]

***Appendix S3: Forest Plots***
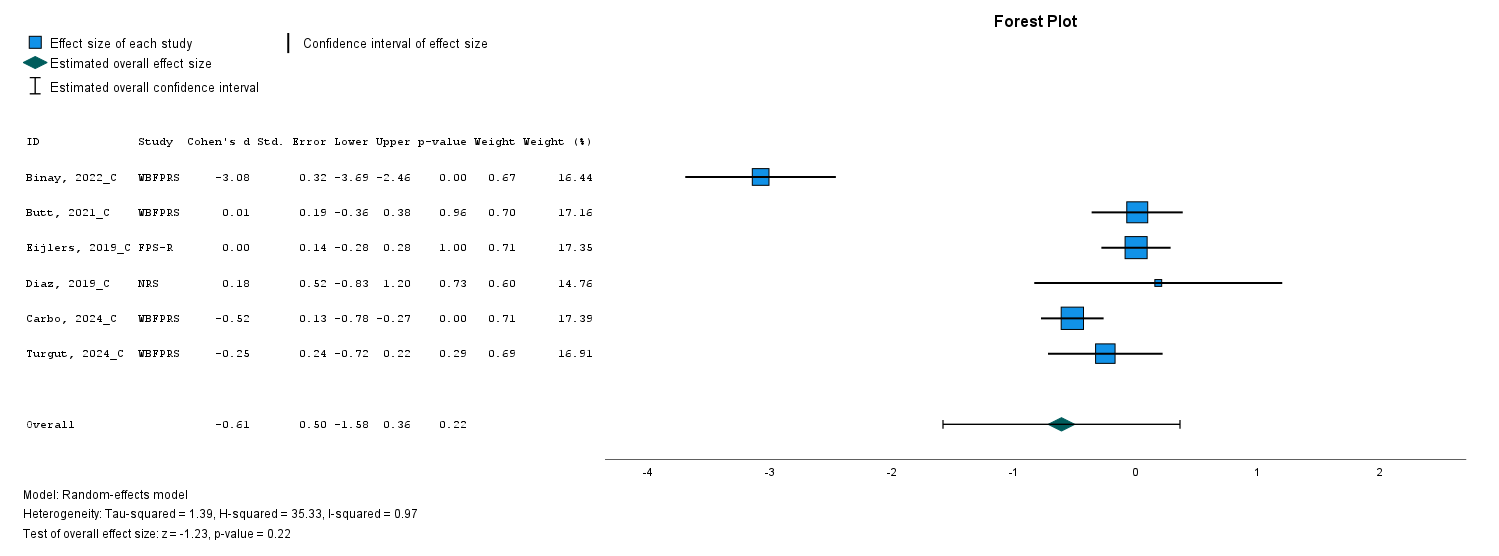


***Figure 2.*** *Random-effects meta-analysis of the effect of XR on acute and postoperative pain as reported by patients, compared to CAU. Pain outcomes were measured using the Wong-Baker Faces Pain Rating Scale, the Faces pain Scale-Revised, Numeric Rating Scale and the Face, Legs, Activity, Cry, Consolability Scale.****Note:***
***C =*** *Child*


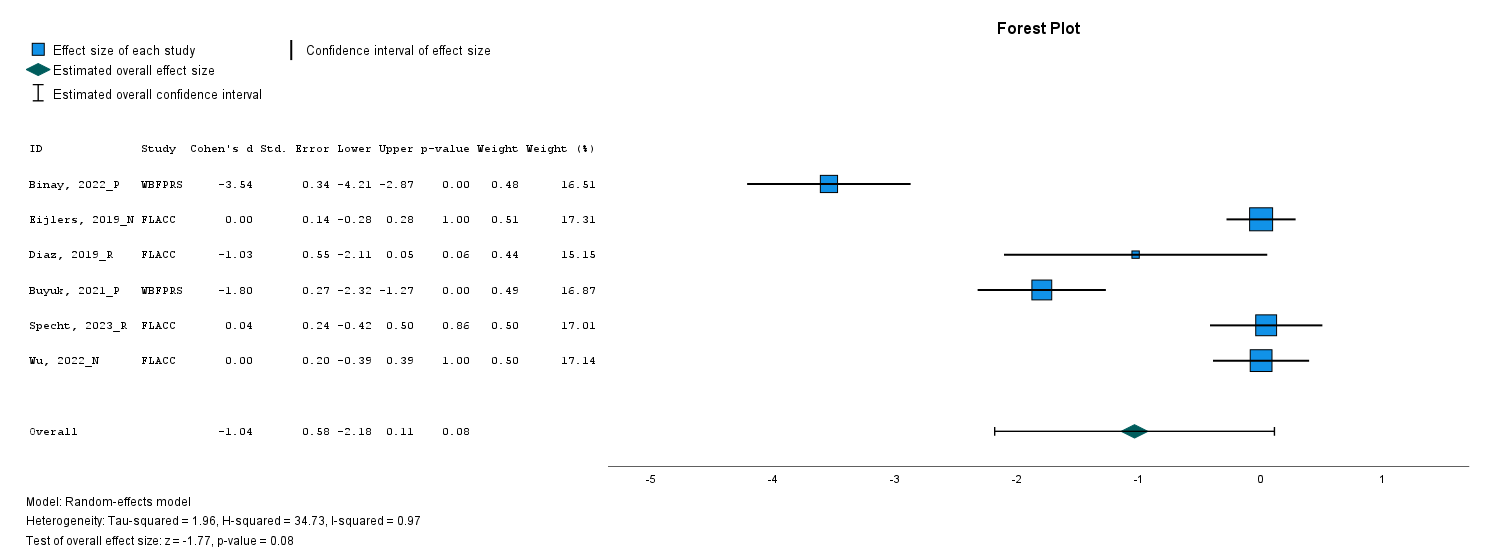


*Figure 3. Random-effects meta-analysis of the effect of XR on acute and postoperative pain as reported by the parents, nurses, or healthcare professionals, compared to CAU. Pain outcomes were measured using the Wong-Baker Faces Pain Rating Scale and the Face, Legs, Activity, Cry, Consolability Scale.
Note:
P = Parent, N = Nurse, R = Researcher.*

***
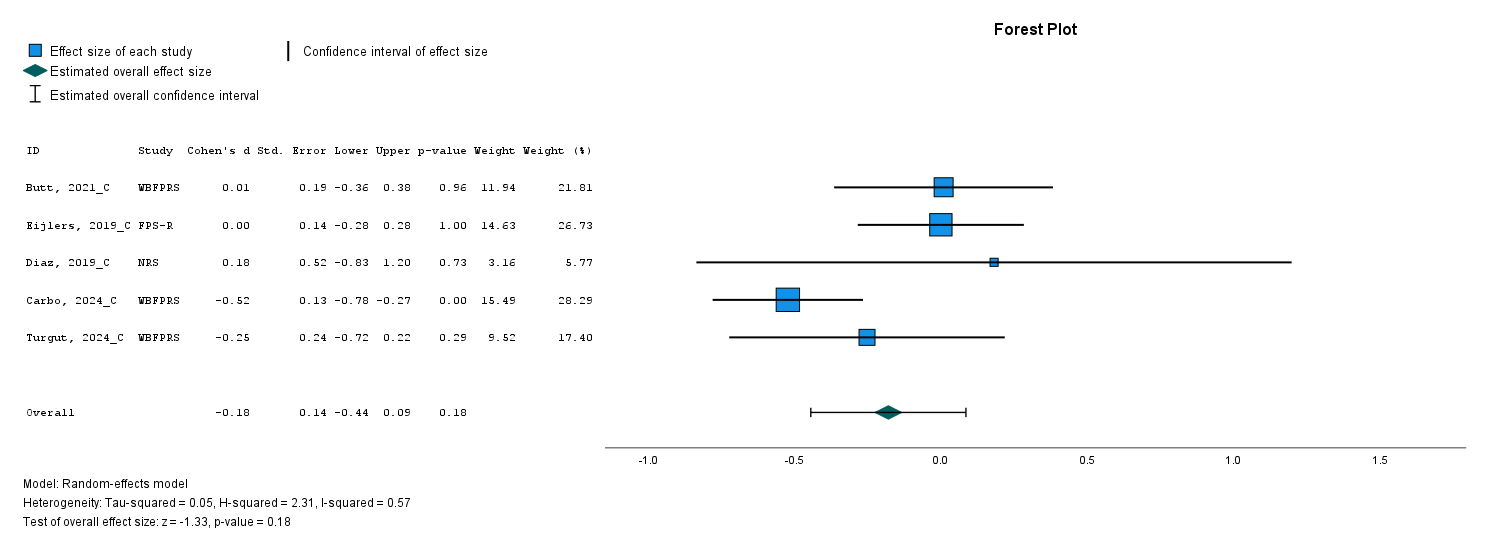
***

***Figure 4.*** *Random-effects meta-analysis of the effect of XR on acute and postoperative pain as reported by patients, compared to CAU, excluding study 23. Pain outcomes were measured using the Wong-Baker Faces Pain Rating Scale, the Faces pain Scale-Revised, Numeric Rating Scale and the Face, Legs, Activity, Cry, Consolability Scale.****Note:***
***C =*** *Child*

*
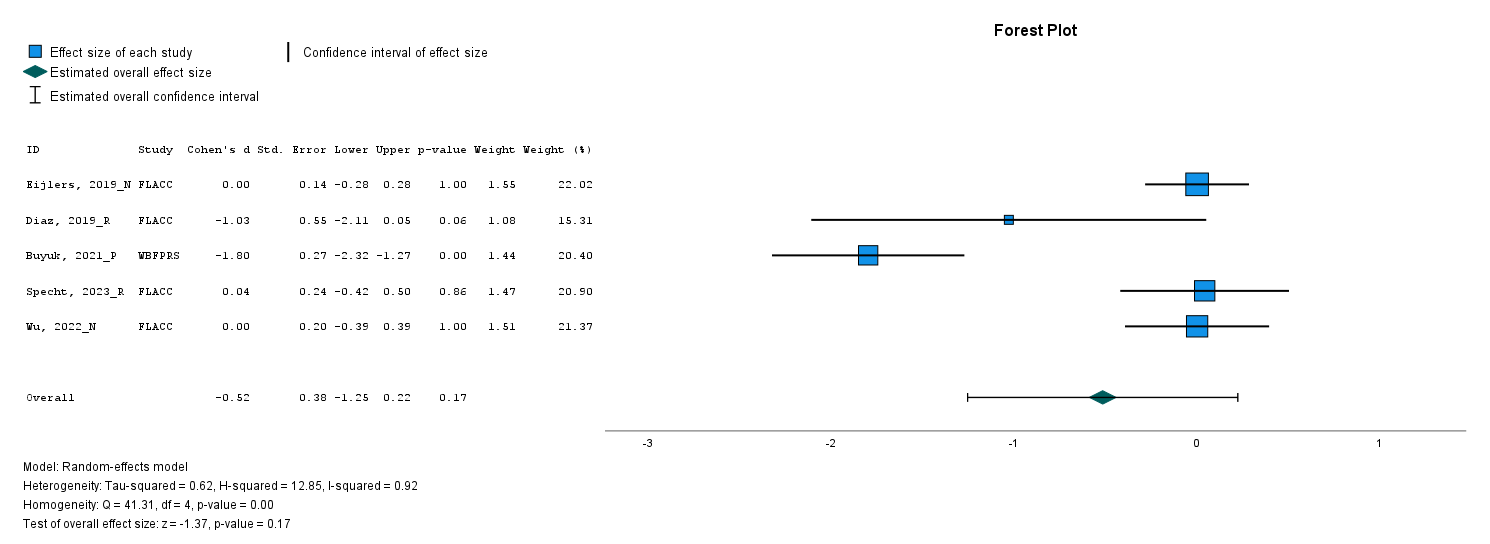
*

*Figure 5. Random-effects meta-analysis of the effect of XR on acute and postoperative pain as reported by the parents, nurses, or healthcare professionals, compared to CAU, excluding study 23. Pain outcomes were measured using the Wong-Baker Faces Pain Rating Scale* *and the Face, Legs, Activity, Cry, Consolability Scale.
Note:
P = Parent, N = Nurse, R = Researcher.*


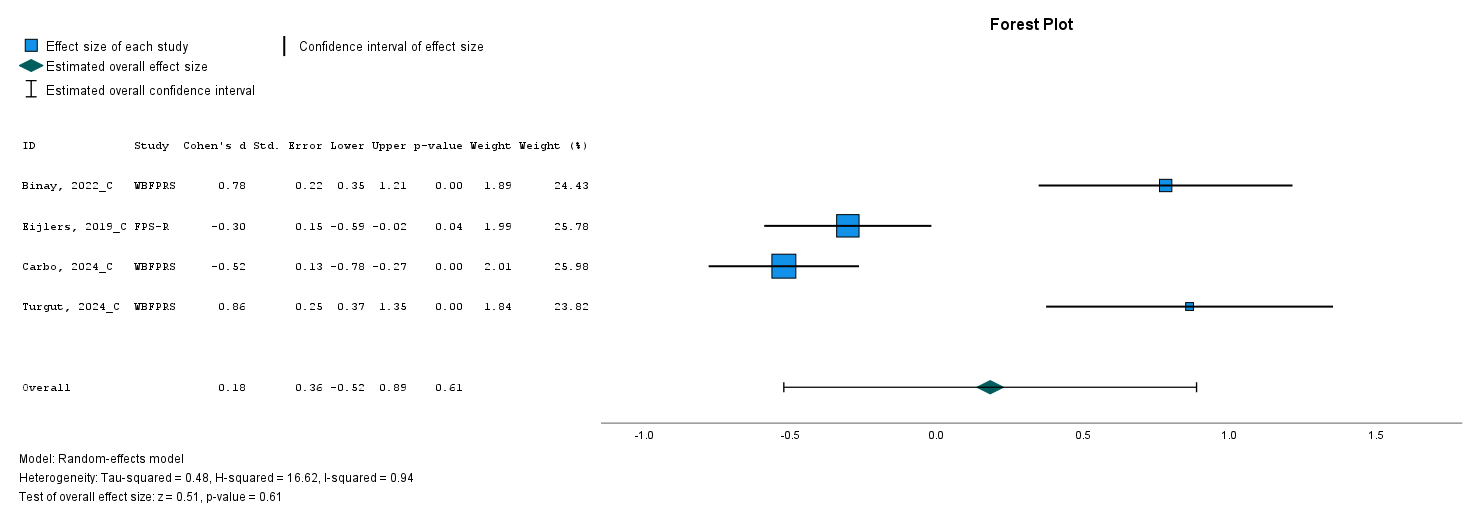

***Figure 6.*** *Random-effects meta-analysis of the effect of XR on postoperative pain as a subgroup reported by patients, compared to CAU. Pain outcomes were measured using the Wong-Baker Faces Pain Rating Scale, the Faces pain Scale-Revised.****Note:***
***C =*** *Child*


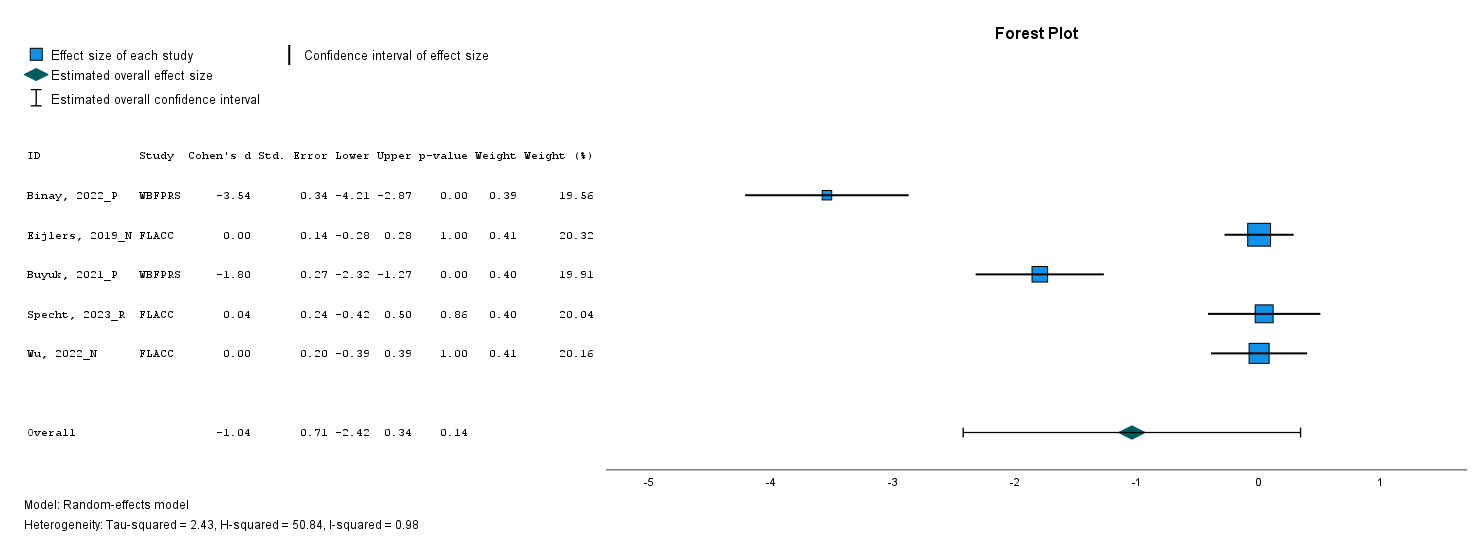


*Figure 7. Random-effects meta-analysis of the effect of XR on postoperative pain as a subgroup reported as reported by the parents, nurses, or healthcare professionals, compared to CAU. Pain outcomes were measured using the Wong-Baker Faces Pain Rating Scale and the Face, Legs, Activity, Cry, Consolability Scale.
Note:
P = Parent, N = Nurse, R = Researcher.*


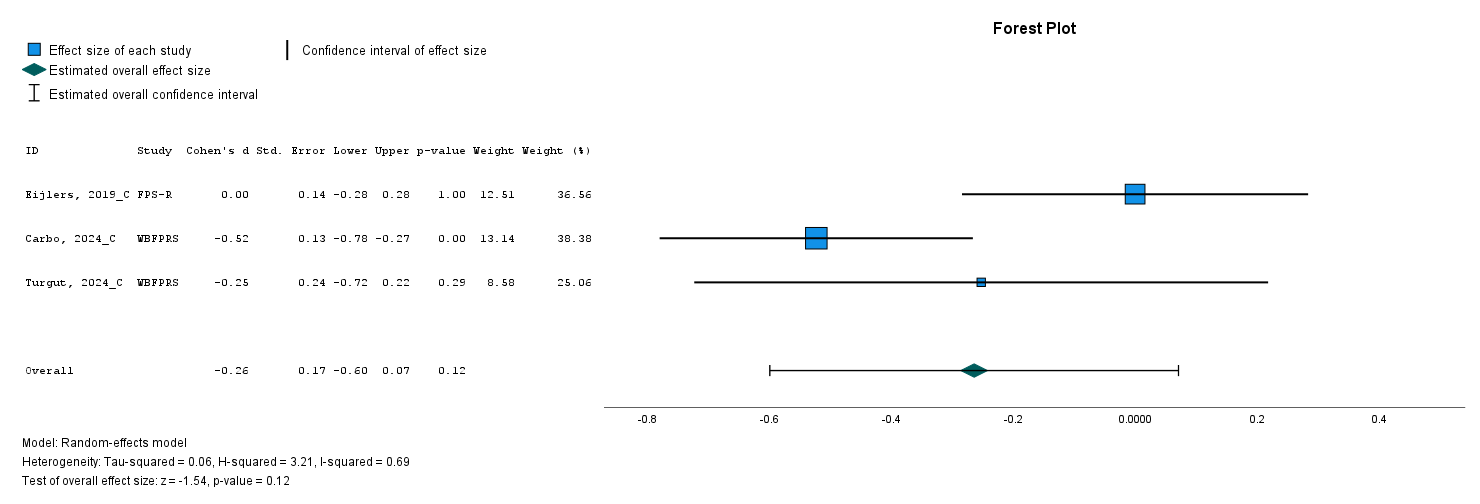
 ***Figure 8.*** *Random-effects meta-analysis of the effect of XR on postoperative pain as a subgroup reported by patients, compared to CAU, excluding study 23. Pain outcomes were measured using the Wong-Baker Faces Pain Rating Scale, the Faces pain Scale-Revised.****Note:***
***C =*** *Child*


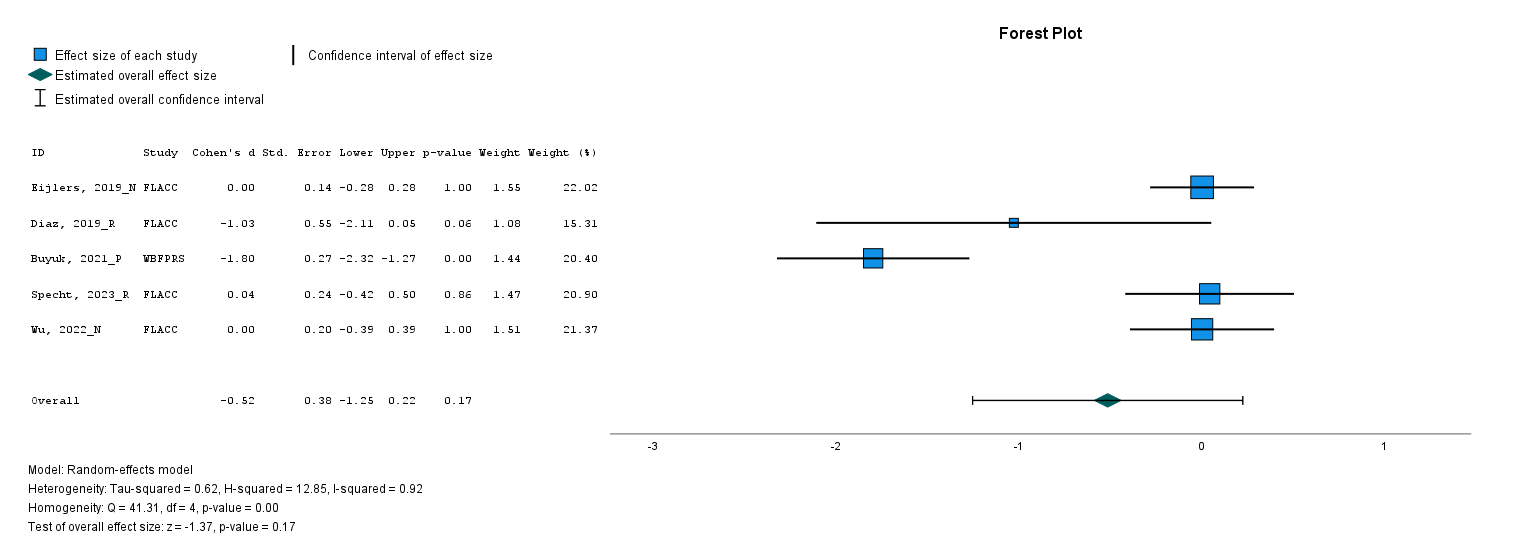


*Figure 9. Random-effects meta-analysis of the effect of XR on postoperative pain as a subgroup reported as reported by the parents, nurses, or healthcare professionals, compared to CAU, excluding study 23. Pain outcomes were measured using the Wong-Baker Faces Pain Rating Scale and the Face, Legs, Activity, Cry, Consolability Scale.
Note:
P = Parent, N = Nurse, R = Researcher.*
