# Supplementary material for: The Analgesic Effect of Extended Reality (XR) on Acute and Postoperative Pain in Children: A Systematic Review and Meta‐Analysis
Source: Paediatr Anaesth. 2026 Mar 7;36(5):479–90. doi: 10.1002/pan.70157 (PMC13054109; doi:10.1002/pan.70157)
Supplement: Supplementary file 4 — Appendix S4: pan70157‐sup‐0004‐AppendixS4.docx. [file PAN-36-479-s004.docx]

***Appendix S4: Funnel Plots***

*
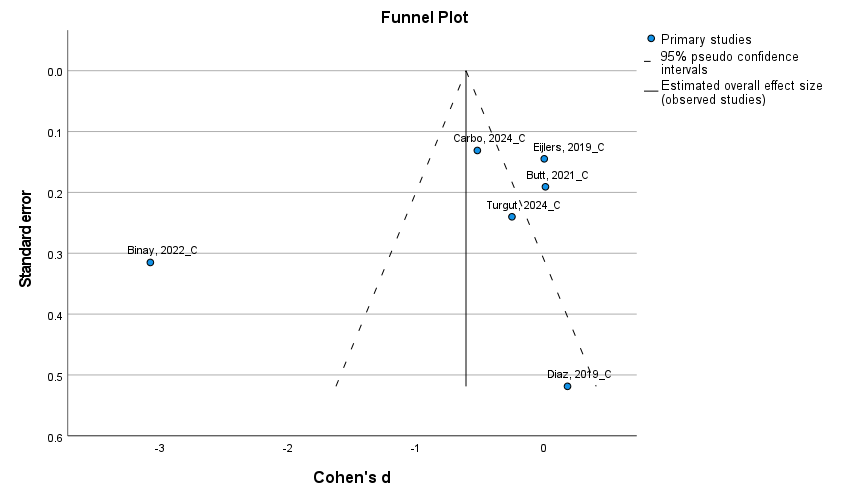
*

***Figure 10. Funnel plot of the primary meta-analysis***

***
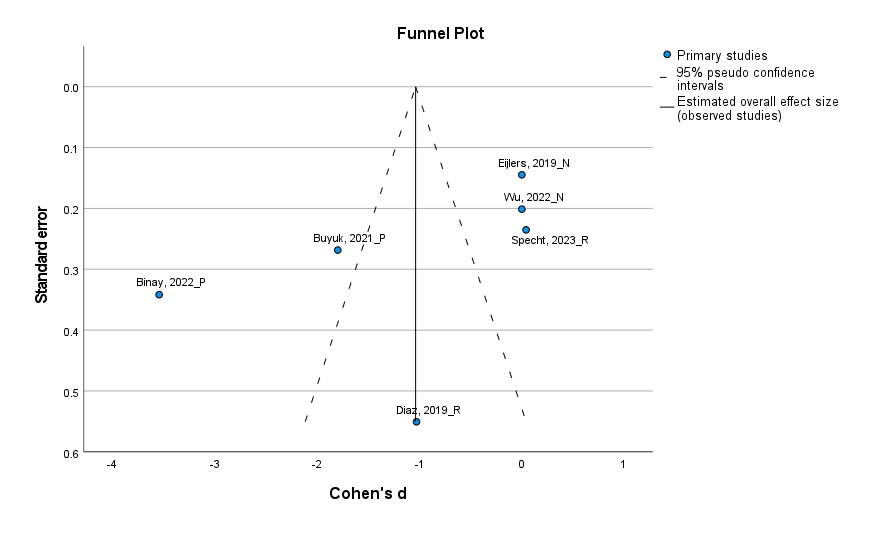

Figure 11. Funnel plot of the secondary meta-analysis***
